# An Innovative Method of Repeated Tie over Dressing for Fixation of skin Graft

**Published:** 2017-05

**Authors:** Shabeer Wani, Ovais Matto, Doaa Andejani, Faris Akmugarian, Faris Aldhagri, Ahmad Wafa

**Affiliations:** Department of Plastic Surgery, King F M C, Riyadh, Saudi Arabia

**Keywords:** Tie over dressing, Skin graft, Keloid, Radiotherapy

**DEAR EDITOR**

Reconstruction of any soft tissue defect is managed as per conventional reconstructive ladder. The options range from healing by secondary intention, primary closure, skin graft and flaps. Skin graft is one of the commonest methods of reconstruction. For proper take of skin graft, the graft needs to be adherent to the wound bed for first few days. The fixation should not allow any hematoma or seroma formation under the graft.^1^ This is achieved by fixing the skin graft with tie over dressing using multiple silk sutures. Repeated tie over dressing is needed in the settings where graft needs to be inspected frequently.^1^

Four patients having recurrent and huge keloids on anterior chest wall in the presternal region were referred from radiation oncology department for possible excision and postoperative radiotherapy. All these patients have previously undergone multiple treatments of their keloids in the form injections and local excisions with out any benefit. The patients were accepted by radiation oncology and were found candidates for immediate radiotherapy after excision of keloid.

In all patients keloids were excised and the soft tissue defect was covered by full thickness skin grafts harvested from groin. The skin grafts were immobilized by tie over dressing in an innovative way. In this method the normal saline bottle used for irrigation was cut at its neck (Figure 1). The skin graft was fixed by cyanoacrylate skin glue and tie over dressing using 2-0 silk sutures. All the patients gave a written informed consent for their inclusion in the study. The identity of all the persons in the study was kept confidential.

**Fig. 1:**
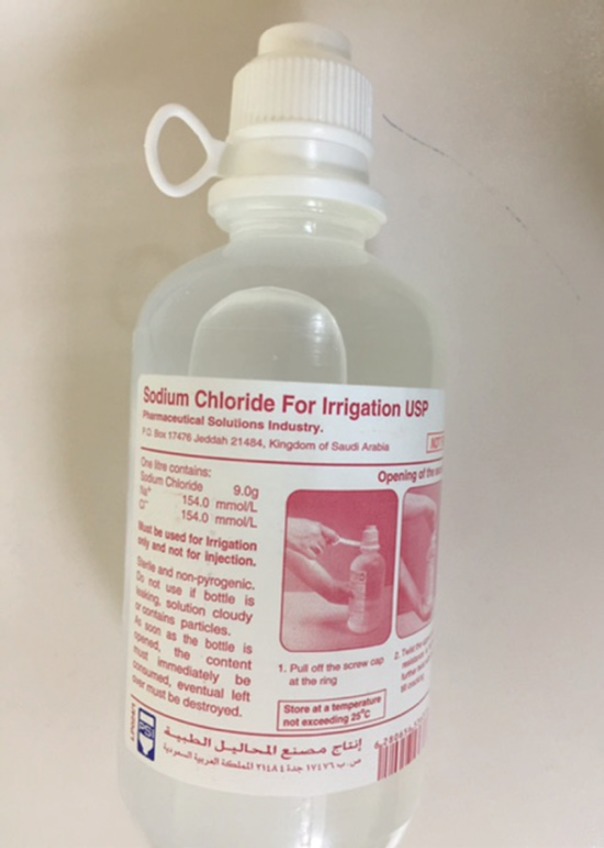
Normal saline bottle for irrigation

Once non adherent layer of gauze and adequate padding is applied on the raw area, the tie over threads were passed from inside out of the cut part of collar of saline bottle and pulled at the appropriate tension to keep the dressing in place (Figure 2). The cap of the bottle was tightened to complete the dressing (Figure 3) ensuring that the graft was maintained in close approximation with the wound surface.

**Fig. 2:**
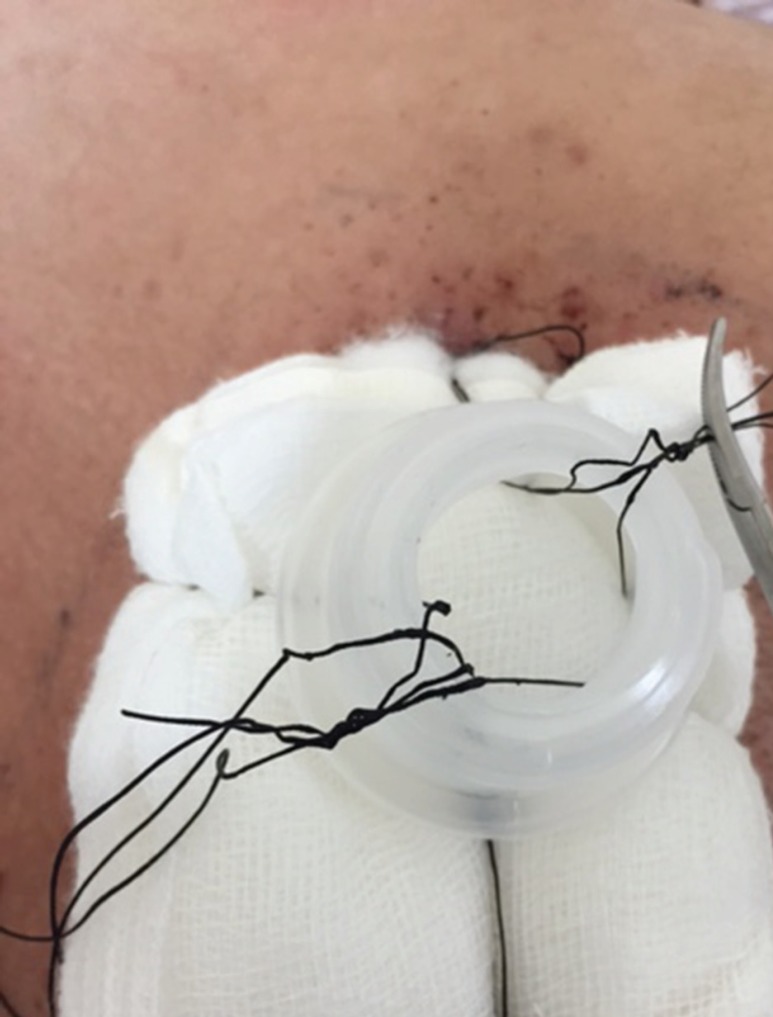
Tie over threads being passed from inside out

**Fig. 3:**
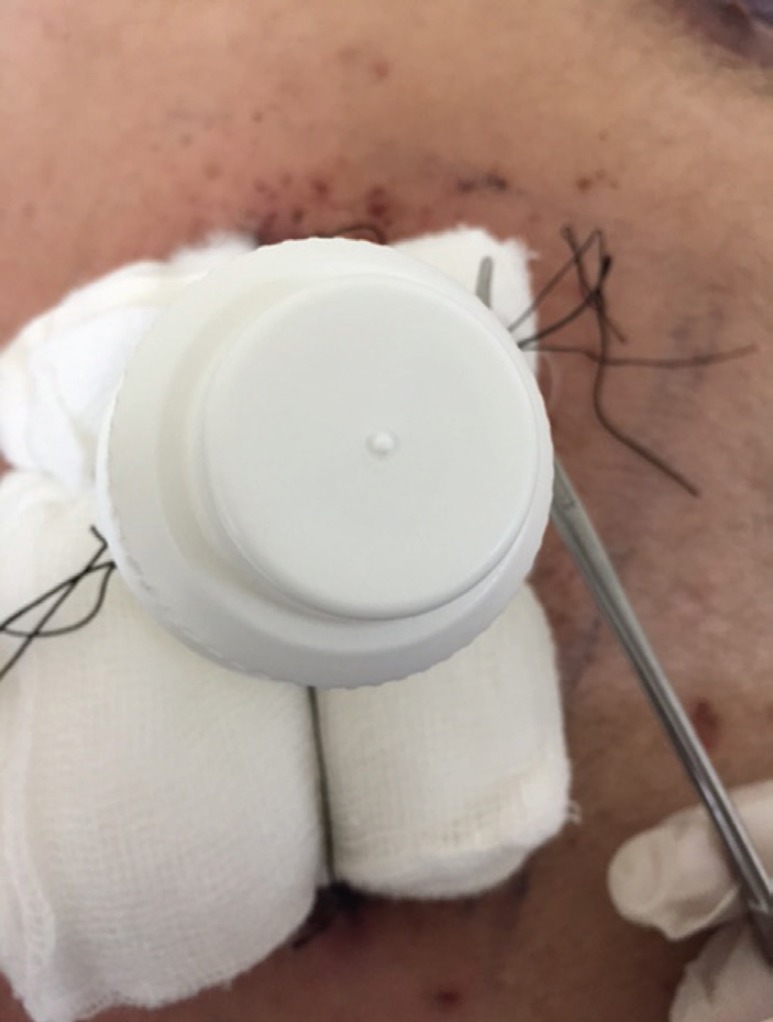
The lid is tightened over the dressing

On postoperative day 1, the dressing is removed by unscrewing the cap and patient is shifted to receive radiotherapy (Figure 4). After finishing the radiotherapy the dressing can be put back and the tie over dressing put on by screwing the cap on the collar (Figure 5). The dressing can be changed repeatedly depending on the requirement, as an outpatient procedure by unscrewing the cap, which can then be easily reapplied. 

**Fig 4:**
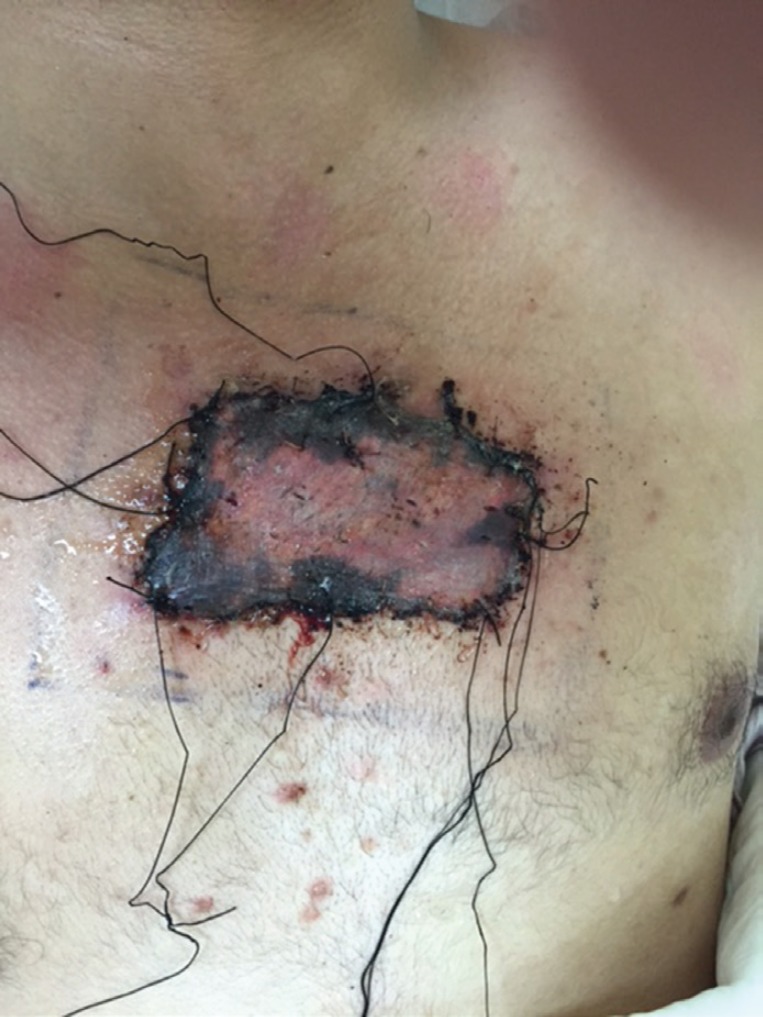
Removal of dressing on postoperative day 1

**Fig. 5:**
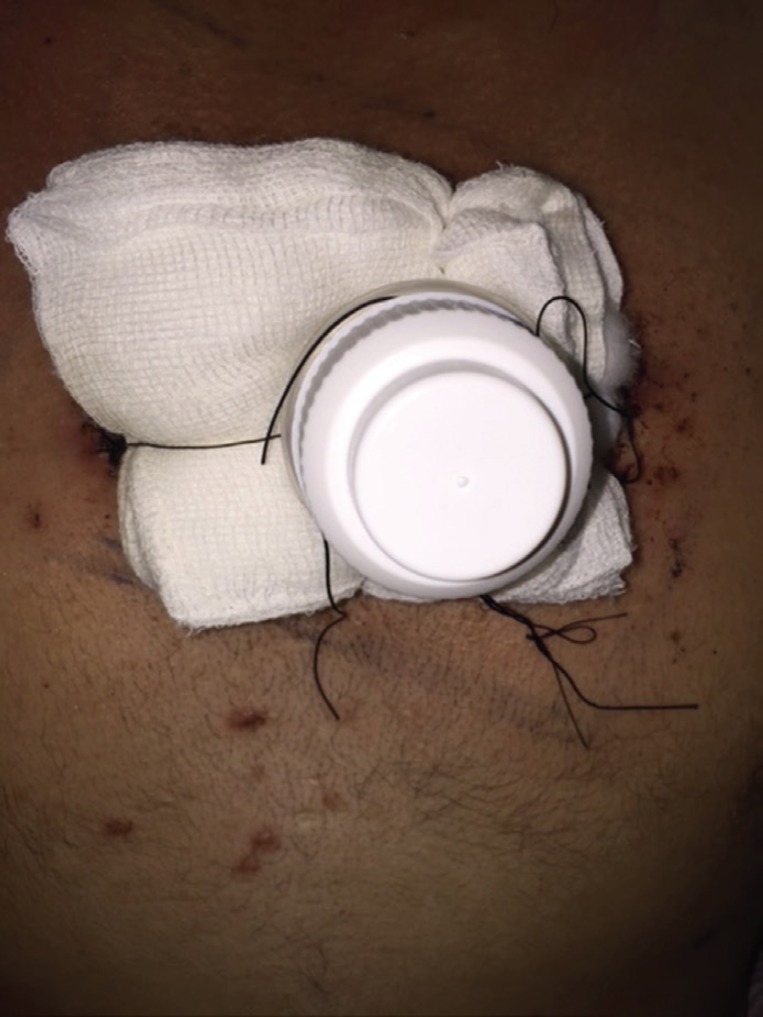
Reapplication of lid after finishing radiotherapy

Skin grafts after applying on wound bed is dressed by applying non-adherent layer of dressing. The dressing can be secured in place by many methods, but can be applied once only. These traditional methods can stabilize the graft till the first dressing post operatively. In some contaminated wounds, the dressing needs to be removed earlier, especially if there is drainage or foul smell.^2^

This approach may also be proper for graft, used to cover defects of some anatomical regions with increases risk of contamination, such as perineal, axillary, and genital or it can be used in areas where base of wound is difficult to immobilize like breast/pectoral region. It is true in cases of our study where repeated dressing is needed for giving local radiotherapy. There are multiple methods that can be used repeatedly for the skin graft stabilization.

Repeated tie over dressings can be done by keeping interrupted sutures long to be used as tie over dressings. These ties over dressing can be made of sutures or rubber bands.^3^ When taking a tie over stitch, both the threads can be left long and only one thread is tied at a time, the other thread is left long for next time.^4^ This dressing can be reapplied only twice. Repeated tie over dressings are also done using bra hooks,^5^ and silk loops.^6^

These techniques are difficult for small dressings especially the bra hooks; the silk loops method is very cumbersome and takes long time to do. Our innovative method being discussed is very fast. It hardly takes 5 min in the hands of a Plastic surgeon to complete the dressing. This dressing technique maintains the advantage of conventional tie over dressing with rapidity and repeativity. Excellent graft take can be expected with appropriate methods of stabilization. We recommend an innovative low cost, simple and rapid method of graft fixation that can be used repeatedly.

## CONFLICT OF INTEREST

The authors declare no conflict of interest.
